# Di­methyl­ammonium tetra­aqua­(hydrogen­sulfato)­sulfato­cuprate(II)

**DOI:** 10.1107/S1600536814004486

**Published:** 2014-03-05

**Authors:** Peter Held

**Affiliations:** a Institut für Kristallographie, Universität zu Köln, Greinstrasse 6, D-50939 Köln, Germany

## Abstract

In the title salt, [(CH_3_)_2_NH_2_][Cu(HSO_4_)(SO_4_)(H_2_O)_4_], one type of cation and anion is present in the asymmetric unit. The Cu^II^ atom in the complex anion, [Cu(HSO_4_)(SO_4_)(H_2_O)_4_]^−^, has a tetra­gonal bipyramidal [4 + 2] coordination caused by a Jahn–Teller distortion, with the aqua ligands in equatorial and two O atoms of tetra­hedral HSO_4_ and SO_4_ units in apical positions. Both types of ions form sheets parallel to (010). The inter­connection within and between the sheets is reinforced by O—H⋯O and N—H⋯O hydrogen bonds, respectively, involving the water mol­ecules, the two types of sulfate anions and the ammonium groups.

## Related literature   

For related structures, see: Montgomery & Lingafelter (1966 ▶); Montgomery *et al.* (1967 ▶); Held (2003 ▶, 2014 ▶). For bond-valence parameters, see: Brown & Altermatt (1985 ▶).
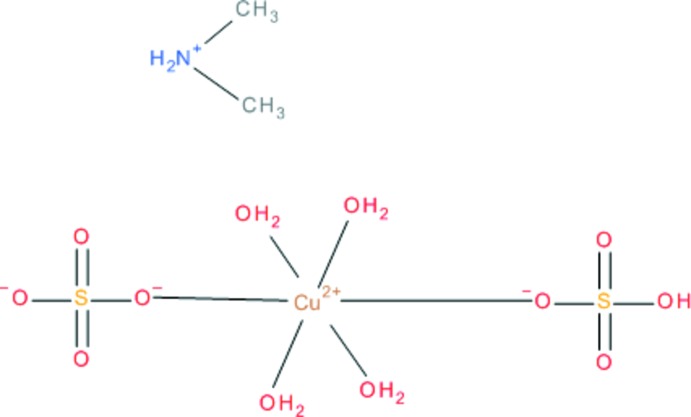


## Experimental   

### 

#### Crystal data  

(C_2_H_8_N)[Cu(HSO_4_)(SO_4_)(H_2_O)_4_]
*M* *_r_* = 374.8
Orthorhombic, 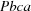

*a* = 7.1825 (9) Å
*b* = 17.9973 (15) Å
*c* = 19.410 (3) Å
*V* = 2509.0 (6) Å^3^
*Z* = 8
Mo *K*α radiation
μ = 2.13 mm^−1^
*T* = 295 K
0.29 × 0.27 × 0.25 mm

#### Data collection  

Nonius MACH3 diffractometer
Absorption correction: ψ scan (North *et al.*, 1968 ▶) *T*_min_ = 0.960, *T*_max_ = 0.999
7482 measured reflections
3801 independent reflections
2231 reflections with *I* > 2σ(*I*)
*R*_int_ = 0.055
3 standard reflections every 100 reflections intensity decay: −1.4%

#### Refinement  

*R*[*F* ^2^ > 2σ(*F* ^2^)] = 0.032
*wR*(*F* ^2^) = 0.092
*S* = 0.97
3801 reflections
196 parameters
8 restraints
H atoms treated by a mixture of independent and constrained refinement
Δρ_max_ = 0.74 e Å^−3^
Δρ_min_ = −0.44 e Å^−3^

### 

Data collection: *CAD-4* (Enraf–Nonius, 1989 ▶); cell refinement: *CAD-4*; data reduction: *WinGX* (Farrugia, 2012 ▶); program(s) used to solve structure: *SIR97* (Altomare *et al.*, 1999 ▶); program(s) used to refine structure: *SHELXL97* (Sheldrick, 2008 ▶); molecular graphics: *ATOMS* (Dowty, 2002 ▶) and *ORTEP-3 for Windows* (Farrugia, 2012 ▶); software used to prepare material for publication: *publCIF* (Westrip, 2010 ▶).

## Supplementary Material

Crystal structure: contains datablock(s) I, global. DOI: 10.1107/S1600536814004486/wm5006sup1.cif

Structure factors: contains datablock(s) I. DOI: 10.1107/S1600536814004486/wm5006Isup2.hkl

CCDC reference: 988936

Additional supporting information:  crystallographic information; 3D view; checkCIF report

## Figures and Tables

**Table 1:** Hydrogen-bond geometry (Å, °)

*D*—H⋯*A*	*D*—H	H⋯*A*	*D*⋯*A*	*D*—H⋯*A*
N3—H3*B*⋯O13^i^	0.90	2.22	2.932 (4)	136
N3—H3*A*⋯O11^ii^	0.90	2.00	2.871 (3)	164
O1—H1*D*⋯O12^iii^	0.84 (2)	1.82 (2)	2.656 (3)	175 (4)
O1—H1*E*⋯O12^iv^	0.85 (2)	1.85 (2)	2.687 (3)	171 (4)
O2—H2*D*⋯O24^v^	0.85 (2)	1.90 (2)	2.745 (3)	174 (3)
O2—H2*E*⋯O24^vi^	0.84 (2)	1.94 (2)	2.777 (3)	174 (4)
O3—H3*D*⋯O23^vii^	0.86 (2)	1.87 (2)	2.722 (3)	173 (4)
O3—H3*E*⋯O14^iv^	0.85 (2)	1.82 (2)	2.661 (3)	167 (4)
O4—H4*D*⋯O23^vi^	0.84 (2)	1.91 (2)	2.753 (3)	175 (4)
O4—H4*E*⋯O14^viii^	0.87 (2)	1.76 (2)	2.627 (3)	171 (5)
O21—H21⋯O13^ix^	0.82	1.71	2.484 (3)	156

Symmetry codes: (i) 
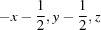
; (ii) 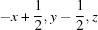; (iii) 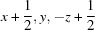; (iv) 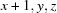; (v) 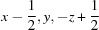; (vi) 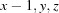; (vii) 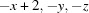; (viii) 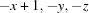; (ix) 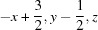.

**Fig. 1.**
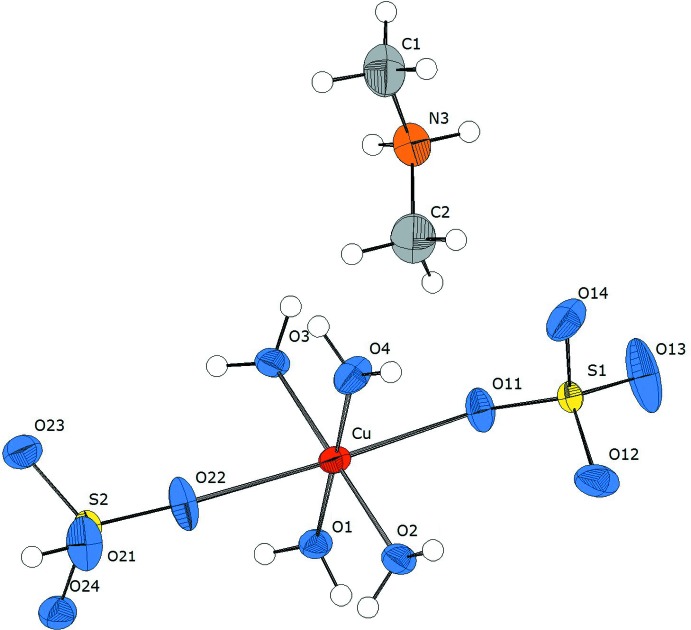
The molecular entities in the structure of the title compound, showing the atom-numbering scheme. Displacement ellipsoids are drawn at the 50% probability level.

**Fig. 2.**
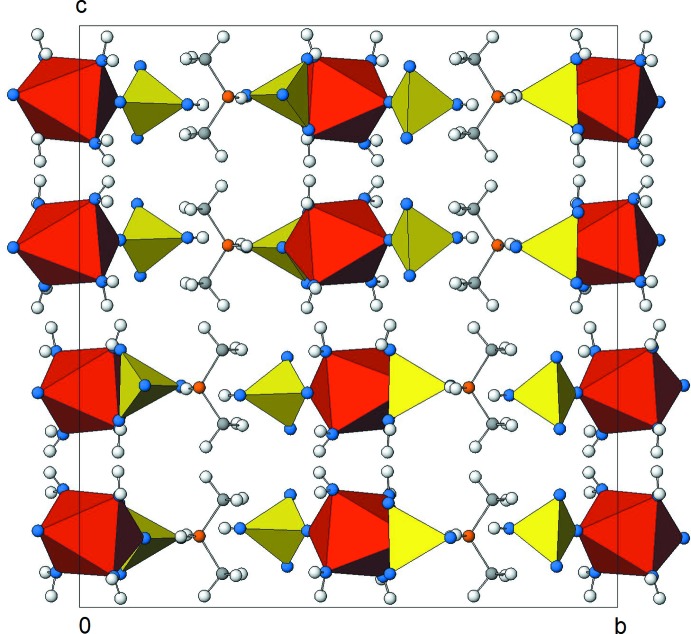
(100)-projection of the crystal structure of the title compound. Colour scheme: [Cu(H2O)4(SO4)(HSO4)] bipyramids (red), (SO4) tetrahedra (yellow), N (orange), C (grey) and H (white).

**Fig. 3.**
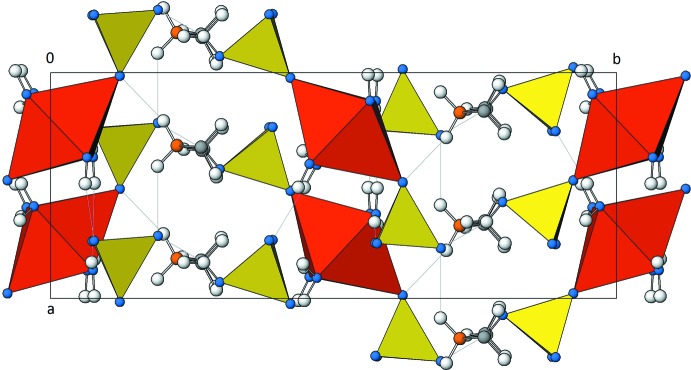
(001)-projection of the crystal structure of the title compound. Colour scheme as in Fig. 2. Hydrogen bonding is indicated by small grey lines.

## supplementary crystallographic information

### 1. Comment

In the course of a systematic search for new "double salts" of simple secondary amines and monovalent cations of various inorganic acids, the structures of the new compounds (C_2_N_2_H_10_)Li_2_(SO_4_)_2_ and [NH_2_(CH_2_CH_3_)_2_][H_2_PO_4_] have been described (Held, 2003, 2014). In continuation of these studies, ethylenediamine and lithium were replaced with dimethylamine and divalent copper, respectively, yielding crystals of the title compound with composition [(CH_3_)_2_NH_2_][Cu(HSO_4_)(SO_4_)(H_2_O)_4_].

The structure of the title compound consists of SO_4_^2-^ and HSO_4_^-^ anions, NH_2_(CH_3_)_2_^+^ and Cu^2+^ cations as well as water molecules as basic structure units. All atoms are located on general Wykoff position 8*c*. The Cu^2+^ cation is surrounded by six O atoms of four equatorially placed water molecules (averaged distance = 1.952 (17) Å) and of two apical sulfate groups (averaged distance = 2.45 (6) Å), forming a distorted tetragonal bipyramid, [Cu(H_2_O)_4_(SO_4_)(HSO_4_)], which is markedly elongated to both apices due to the Jahn-Teller-effect of the Cu^2+^ cation, leading to a pronounced [4 + 2] coordination (Fig. 1) and an overall bond valence sum (Brown & Altermatt, 1985) of 2.17 valence units. Caused by the dissimilar coordination partners, the Cu—O distances vary widely in comparison with more uniform O environments, *e.g.* in the Tutton's salt (NH_4_)_2_Cu(SO_4_)_2_(H_2_O)_6_ with a hexa-coordination of Cu^2+^ by water molecules (Montgomery & Lingafelter (1966); Montgomery *et al.*(1967)). As expected, the S—O distance of the OH-function (1.545 (2) Å) is considerably longer than the other S—O distances (average distance 1.459 (11) Å).

In the title compound, the [Cu(H_2_O)_4_Cu(SO_4_)_2_]^2-^ anions form sheets parallel to (010), hold apart from each other by dimethylammonium groups (Fig. 2). Hydrogen bonds of medium strength involving water molecules as donor groups and O atoms of the sulfate anions as acceptor groups interconnect neighbouring [Cu(H_2_O)_4_(SO_4_)(HSO_4_)]^2-^ units. Together with N—H···O hydrogen bonds of the ammonium hydrogen atoms, a three-dimensional framework (Fig. 3) is formed.

### 2. Experimental

The title compound was obtained by reaction of aqueous solution of copper(II) sulfate with dimethylamine and sulfuric acid in the stoichiometric ratio 1:1:1. The solution was kept at room temperature by cooling. The title compound crystallized by slow evaporation of the solvent at room temperature in form of optical clear, light-blue crystals with dimensions up to 5 mm within a few weeks.

### 3. Refinement

The H atoms were clearly discernible from difference Fourier maps. However, to all hydrogen atoms riding model contraints were applied in the least squares refinement, with C—H = 0.96 Å for methyl H atoms (*U*_iso_(H) = 1.5*U*_eq_(C)), with N—H = 0.90 Å (*U*_iso_(H) = 1.2*U*_eq_(N)) and with O—H = 0.82 Å (*U*_iso_(H) = 1.2*U*_eq_(O)) for the H atom of the HSO_4_^-^ anion . All H atoms of the water molecules were refined with a distance restraint of O—H ≈ 0.87 Å.

### Crystal data

**Table:**

(C_2_H_8_N)[Cu(HSO_4_)(SO_4_)(H_2_O)_4_]	*F*(000) = 1544
*M**_r_* = 374.8	*D*_x_ = 1.985 Mg m^−^^3^
Orthorhombic, *P**b**c**a*	Mo *K*α radiation, λ = 0.71073 Å
Hall symbol: -P 2ac 2ab	Cell parameters from 25 reflections
*a* = 7.1825 (9) Å	θ = 21.0–26.0°
*b* = 17.9973 (15) Å	µ = 2.13 mm^−^^1^
*c* = 19.410 (3) Å	*T* = 295 K
*V* = 2509.0 (6) Å^3^	Parallelepiped, light blue
*Z* = 8	0.29 × 0.27 × 0.25 mm

### Data collection

**Table:**

Nonius MACH3 diffractometer	2231 reflections with *I* > 2σ(*I*)
Radiation source: fine-focus sealed tube	*R*_int_ = 0.055
Graphite monochromator	θ_max_ = 30.4°, θ_min_ = 2.3°
ω/2θ scans	*h* = −10→0
Absorption correction: ψ scan (North *et al.*, 1968)	*k* = −25→0
*T*_min_ = 0.960, *T*_max_ = 0.999	*l* = −27→27
7482 measured reflections	3 standard reflections every 100 reflections
3801 independent reflections	intensity decay: −1.4%

### Refinement

**Table:**

Refinement on *F*^2^	Secondary atom site location: difference Fourier map
Least-squares matrix: full	Hydrogen site location: inferred from neighbouring sites
*R*[*F*^2^ > 2σ(*F*^2^)] = 0.032	H atoms treated by a mixture of independent and constrained refinement
*wR*(*F*^2^) = 0.092	*w* = 1/[σ^2^(*F*_o_^2^) + (0.0423*P*)^2^ + 0.4413*P*] where *P* = (*F*_o_^2^ + 2*F*_c_^2^)/3
*S* = 0.97	(Δ/σ)_max_ = 0.001
3801 reflections	Δρ_max_ = 0.74 e Å^−^^3^
196 parameters	Δρ_min_ = −0.44 e Å^−^^3^
8 restraints	Extinction correction: *SHELXL97* (Sheldrick, 2008), Fc^*^=kFc[1+0.001xFc^2^λ^3^/sin(2θ)]^-1/4^
Primary atom site location: structure-invariant direct methods	Extinction coefficient: 0.0081 (3)

### Special details

**Table:**

Experimental. A suitable single-crystal was carefully selected under a polarizing microscope and mounted in a glass capillary.
Geometry. All e.s.d.'s (except the e.s.d. in the dihedral angle between two l.s. planes) are estimated using the full covariance matrix. The cell e.s.d.'s are taken into account individually in the estimation of e.s.d.'s in distances, angles and torsion angles; correlations between e.s.d.'s in cell parameters are only used when they are defined by crystal symmetry. An approximate (isotropic) treatment of cell e.s.d.'s is used for estimating e.s.d.'s involving l.s. planes.
Refinement. Refinement of *F*^2^ against ALL reflections. The weighted *R*-factor *wR* and goodness of fit *S* are based on *F*^2^, conventional *R*-factors *R* are based on *F*, with *F* set to zero for negative *F*^2^. The threshold expression of *F*^2^ > σ(*F*^2^) is used only for calculating *R*-factors(gt) *etc*. and is not relevant to the choice of reflections for refinement. *R*-factors based on *F*^2^ are statistically about twice as large as those based on *F*, and *R*- factors based on ALL data will be even larger.

### Fractional atomic coordinates and isotropic or equivalent isotropic displacement parameters (Å^2^)

**Table:**

	*x*	*y*	*z*	*U*_iso_*/*U*_eq_	
Cu	0.73284 (4)	0.015242 (18)	0.126573 (17)	0.01956 (10)	
O1	0.8740 (3)	0.07478 (12)	0.19257 (11)	0.0228 (4)	
O2	0.5919 (3)	−0.02976 (12)	0.20235 (11)	0.0253 (5)	
O3	0.8752 (3)	0.06422 (15)	0.05293 (11)	0.0293 (5)	
O4	0.5959 (3)	−0.04258 (14)	0.06014 (12)	0.0312 (5)	
S1	0.31024 (8)	0.11572 (4)	0.11869 (4)	0.01745 (14)	
O11	0.5141 (3)	0.12213 (11)	0.11860 (11)	0.0278 (5)	
O12	0.2463 (3)	0.07405 (15)	0.17900 (12)	0.0422 (6)	
O13	0.2226 (3)	0.18941 (12)	0.11961 (17)	0.0566 (9)	
O14	0.2441 (3)	0.07687 (16)	0.05733 (11)	0.0425 (7)	
S2	1.14451 (9)	−0.12021 (4)	0.13213 (4)	0.01923 (15)	
O21	1.0717 (3)	−0.20091 (11)	0.13532 (13)	0.0376 (6)	
H21	1.1602	−0.2297	0.1359	0.056*	
O22	0.9771 (3)	−0.07646 (12)	0.13129 (14)	0.0397 (6)	
O23	1.2575 (3)	−0.11363 (13)	0.07020 (10)	0.0326 (5)	
O24	1.2594 (3)	−0.10790 (13)	0.19321 (10)	0.0305 (5)	
N3	−0.3234 (4)	−0.27683 (14)	0.12215 (14)	0.0310 (6)	
H3A	−0.2138	−0.3010	0.1241	0.037*	
H3B	−0.4141	−0.3113	0.1212	0.037*	
C1	−0.3443 (6)	−0.2325 (2)	0.1854 (2)	0.0477 (10)	
H1A	−0.3395	−0.2647	0.2248	0.072*	
H1B	−0.4617	−0.2070	0.1845	0.072*	
H1C	−0.2452	−0.1968	0.1881	0.072*	
C2	−0.3304 (6)	−0.2343 (2)	0.0576 (2)	0.0465 (10)	
H2A	−0.3167	−0.2676	0.0192	0.070*	
H2B	−0.2312	−0.1986	0.0570	0.070*	
H2C	−0.4478	−0.2091	0.0543	0.070*	
H1D	0.841 (5)	0.0743 (19)	0.2340 (11)	0.044 (12)*	
H1E	0.992 (3)	0.075 (2)	0.193 (2)	0.052 (14)*	
H2D	0.651 (4)	−0.0538 (16)	0.2328 (13)	0.026 (9)*	
H2E	0.489 (4)	−0.051 (2)	0.198 (2)	0.053 (13)*	
H3D	0.827 (5)	0.077 (2)	0.0141 (13)	0.056 (13)*	
H3E	0.994 (3)	0.063 (2)	0.050 (2)	0.040 (11)*	
H4D	0.490 (3)	−0.062 (2)	0.062 (2)	0.058 (14)*	
H4E	0.637 (6)	−0.054 (2)	0.0194 (13)	0.072 (15)*	

### Atomic displacement parameters (Å^2^)

**Table:**

	*U*^11^	*U*^22^	*U*^33^	*U*^12^	*U*^13^	*U*^23^
Cu	0.01968 (17)	0.02313 (17)	0.01587 (14)	−0.00449 (13)	0.00006 (14)	0.00032 (16)
O1	0.0208 (11)	0.0291 (11)	0.0184 (10)	−0.0035 (9)	−0.0013 (8)	−0.0016 (9)
O2	0.0204 (11)	0.0325 (13)	0.0230 (10)	−0.0041 (10)	−0.0013 (8)	0.0079 (9)
O3	0.0179 (11)	0.0488 (15)	0.0212 (10)	−0.0047 (11)	−0.0006 (9)	0.0110 (10)
O4	0.0244 (12)	0.0445 (14)	0.0247 (11)	−0.0111 (11)	0.0019 (9)	−0.0131 (10)
S1	0.0138 (3)	0.0171 (3)	0.0214 (3)	0.0002 (2)	−0.0004 (2)	−0.0004 (3)
O11	0.0145 (8)	0.0256 (10)	0.0433 (13)	−0.0021 (7)	−0.0001 (9)	0.0009 (10)
O12	0.0254 (12)	0.0729 (19)	0.0283 (11)	−0.0128 (13)	−0.0034 (10)	0.0189 (12)
O13	0.0242 (11)	0.0190 (10)	0.127 (3)	0.0033 (9)	−0.0063 (16)	−0.0017 (15)
O14	0.0236 (11)	0.0750 (19)	0.0288 (11)	−0.0113 (13)	0.0038 (10)	−0.0206 (12)
S2	0.0168 (3)	0.0172 (3)	0.0237 (3)	0.0008 (2)	−0.0003 (3)	−0.0003 (3)
O21	0.0240 (10)	0.0198 (10)	0.0689 (16)	−0.0024 (8)	0.0041 (11)	−0.0010 (11)
O22	0.0227 (10)	0.0305 (12)	0.0658 (16)	0.0115 (9)	−0.0030 (12)	−0.0021 (13)
O23	0.0296 (11)	0.0438 (13)	0.0244 (10)	−0.0043 (12)	0.0014 (9)	0.0018 (9)
O24	0.0281 (11)	0.0400 (12)	0.0233 (10)	−0.0026 (11)	−0.0024 (9)	−0.0032 (9)
N3	0.0294 (12)	0.0226 (11)	0.0411 (15)	0.0025 (10)	0.0030 (12)	−0.0047 (12)
C1	0.037 (2)	0.053 (2)	0.053 (2)	−0.0009 (19)	0.0071 (18)	−0.023 (2)
C2	0.046 (3)	0.039 (2)	0.055 (2)	−0.0008 (19)	−0.0074 (19)	0.0110 (19)

### Geometric parameters (Å, º)

**Table:**

Cu—O4	1.927 (2)	S1—O11	1.4689 (19)
Cu—O1	1.954 (2)	S2—O22	1.438 (2)
Cu—O2	1.961 (2)	S2—O23	1.455 (2)
Cu—O3	1.966 (2)	S2—O24	1.461 (2)
Cu—O22	2.410 (2)	S2—O21	1.545 (2)
Cu—O11	2.489 (2)	O21—H21	0.8200
O1—H1D	0.838 (18)	N3—C2	1.470 (5)
O1—H1E	0.846 (18)	N3—C1	1.471 (4)
O2—H2D	0.848 (18)	N3—H3A	0.9000
O2—H2E	0.837 (19)	N3—H3B	0.9000
O3—H3D	0.857 (18)	C1—H1A	0.9600
O3—H3E	0.852 (18)	C1—H1B	0.9600
O4—H4D	0.843 (18)	C1—H1C	0.9600
O4—H4E	0.869 (19)	C2—H2A	0.9600
S1—O14	1.461 (2)	C2—H2B	0.9600
S1—O12	1.464 (2)	C2—H2C	0.9600
S1—O13	1.468 (2)		
			
O4—Cu—O1	178.97 (10)	O14—S1—O11	111.16 (14)
O4—Cu—O2	90.88 (10)	O12—S1—O11	110.73 (14)
O1—Cu—O2	90.15 (9)	O13—S1—O11	110.88 (13)
O4—Cu—O3	91.21 (11)	S1—O11—Cu	124.65 (12)
O1—Cu—O3	87.76 (9)	O22—S2—O23	114.37 (15)
O2—Cu—O3	177.57 (10)	O22—S2—O24	113.48 (15)
O4—Cu—O22	91.55 (10)	O23—S2—O24	110.06 (12)
O1—Cu—O22	88.45 (9)	O22—S2—O21	103.44 (13)
O2—Cu—O22	93.69 (9)	O23—S2—O21	107.35 (14)
O3—Cu—O22	87.49 (10)	O24—S2—O21	107.53 (14)
O4—Cu—O11	93.07 (9)	S2—O21—H21	109.5
O1—Cu—O11	86.81 (8)	S2—O22—Cu	169.84 (15)
O2—Cu—O11	92.29 (8)	C2—N3—C1	115.2 (3)
O3—Cu—O11	86.36 (9)	C2—N3—H3A	108.5
O22—Cu—O11	172.37 (7)	C1—N3—H3A	108.5
Cu—O1—H1D	118 (3)	C2—N3—H3B	108.5
Cu—O1—H1E	122 (3)	C1—N3—H3B	108.5
H1D—O1—H1E	106 (4)	H3A—N3—H3B	107.5
Cu—O2—H2D	118 (2)	N3—C1—H1A	109.5
Cu—O2—H2E	125 (3)	N3—C1—H1B	109.5
H2D—O2—H2E	106 (4)	H1A—C1—H1B	109.5
Cu—O3—H3D	123 (3)	N3—C1—H1C	109.5
Cu—O3—H3E	123 (3)	H1A—C1—H1C	109.5
H3D—O3—H3E	111 (4)	H1B—C1—H1C	109.5
Cu—O4—H4D	132 (3)	N3—C2—H2A	109.5
Cu—O4—H4E	124 (3)	N3—C2—H2B	109.5
H4D—O4—H4E	104 (4)	H2A—C2—H2B	109.5
O14—S1—O12	107.74 (15)	N3—C2—H2C	109.5
O14—S1—O13	107.63 (17)	H2A—C2—H2C	109.5
O12—S1—O13	108.58 (17)	H2B—C2—H2C	109.5

### Hydrogen-bond geometry (Å, º)

**Table:**

*D*—H···*A*	*D*—H	H···*A*	*D*···*A*	*D*—H···*A*
N3—H3*B*···O13^i^	0.90	2.22	2.932 (4)	136
N3—H3*A*···O11^ii^	0.90	2.00	2.871 (3)	164
O1—H1*D*···O12^iii^	0.84 (2)	1.82 (2)	2.656 (3)	175 (4)
O1—H1*E*···O12^iv^	0.85 (2)	1.85 (2)	2.687 (3)	171 (4)
O2—H2*D*···O24^v^	0.85 (2)	1.90 (2)	2.745 (3)	174 (3)
O2—H2*E*···O24^vi^	0.84 (2)	1.94 (2)	2.777 (3)	174 (4)
O3—H3*D*···O23^vii^	0.86 (2)	1.87 (2)	2.722 (3)	173 (4)
O3—H3*E*···O14^iv^	0.85 (2)	1.82 (2)	2.661 (3)	167 (4)
O4—H4*D*···O23^vi^	0.84 (2)	1.91 (2)	2.753 (3)	175 (4)
O4—H4*E*···O14^viii^	0.87 (2)	1.76 (2)	2.627 (3)	171 (5)
O21—H21···O13^ix^	0.82	1.71	2.484 (3)	156

Symmetry codes: (i) −*x*−1/2, *y*−1/2, *z*; (ii) −*x*+1/2, *y*−1/2, *z*; (iii) *x*+1/2, *y*, −*z*+1/2; (iv) *x*+1, *y*, *z*; (v) *x*−1/2, *y*, −*z*+1/2; (vi) *x*−1, *y*, *z*; (vii) −*x*+2, −*y*, −*z*; (viii) −*x*+1, −*y*, −*z*; (ix) −*x*+3/2, *y*−1/2, *z*.
